# Evaluation of Clinical and Radiological Outcomes of Rigid, Dynamic, and Hybrid Stabilization Systems in Degenerative Lumbar Spine Surgery

**DOI:** 10.3390/jcm15155880

**Published:** 2026-07-28

**Authors:** Ibrahim Taha Albas, Uzay Erdogan, Gurkan Berikol, Furkan Almas, Mehmet Yigit Akgun, Ozkan Ates, Tunc Oktenoglu, Ali Fahir Ozer

**Affiliations:** 1 Department of Neurosurgery, Bakırkoy Prof. Dr. Mazhar Osman Training and Research Hospital, 34147 Istanbul, Turkey; 2 Department of Neurosurgery, Sincan Training and Research Hospital, Health Sciences University, 06949 Ankara, Turkey; 3 Department of Neurosurgery, Koc University Hospital, 34010 Istanbul, Turkey; 4 Spine Center, Koc University Hospital, 34010 Istanbul, Turkey; 5 Biomechanics and Endurance Laboratory, Koc University Research Center for Translational Medicine, 34010 Istanbul, Turkey

**Keywords:** adjacent segment disease, degenerative lumbar spine, dynamic stabilization, hybrid system, PEEK rod, transforaminal lumbar interbody fusion (TLIF)

## Abstract

**Objectives**: Retrospective comparative cohort study. This study aimed to compare the clinical and radiological outcomes of rigid, dynamic, and hybrid stabilization systems in the surgical management of degenerative lumbar spine disease, with a focus on adjacent segment disease (ASD), fusion-related complications, and postoperative recovery. Rigid fusion techniques provide immediate stability but may increase mechanical stress at adjacent segments, leading to ASD. Dynamic and hybrid constructs aim to preserve more physiological motion, potentially reducing fusion-related complications. **Methods**: A retrospective analysis was conducted on 86 patients who underwent posterior lumbar stabilization between January 2017 and December 2021. Patients were categorized into four groups based on the surgical technique: Rigid (titanium rod), Dynamic (PEEK rod), Rigid + TLIF (Transforaminal Lumbar Interbody Fusion), and Hybrid (Dynamic + TLIF). Clinical evaluation included the Visual Analog Scale (VAS) for back and leg pain. Radiological assessments comprised segmental and total range of motion (ROM), disc and foraminal height, fusion success, pseudoarthrosis, and the presence of ASD. **Results**: All surgical approaches resulted in significant postoperative improvement in back and leg pain (*p* < 0.001). The incidence of ASD was highest in the Rigid + TLIF group (45.5%, *p* < 0.001) and lowest in the Dynamic (4.8%) and Hybrid (4.2%) groups. Pseudoarthrosis was most frequently observed in the Rigid group (26.3%). The Hybrid group demonstrated favorable outcomes, with a high anterior fusion rate (91.7%) and low rates of ASD and pseudoarthrosis. **Conclusions**: All stabilization techniques were associated with significant postoperative clinical improvement. In this retrospective cohort, dynamic and hybrid constructs showed lower observed rates of early symptomatic ASD than the Rigid + TLIF construct, while TLIF-treated groups showed higher anterior fusion rates. Because treatment allocation was nonrandomized and adjustment for all potential confounders was not possible, these findings demonstrate associations rather than causal treatment effects. Larger prospective studies with longer follow-up are required to determine whether the stabilization strategy independently influences ASD, fusion, or pseudoarthrosis.

## 1. Introduction

Degenerative lumbar spine disorders—including lumbar disc herniation, spinal stenosis, lateral recess syndrome, and spondylolisthesis—are common conditions that can result in low back pain, radiculopathy, and neurological deficits, ultimately impairing quality of life. When conservative treatments such as pharmacotherapy, physical therapy, and epidural injections fail to provide sufficient relief, surgical intervention becomes necessary. In patients with radiographic or clinical signs of segmental instability, posterior stabilization with instrumentation remains the gold-standard approach.

Conventional surgical treatment typically involves spinal fusion with transpedicular screws and rigid rods composed of titanium alloys. This technique aims to relieve pain and stabilize the affected segment. However, a well-documented complication of rigid fusion is adjacent segment disease (ASD), a condition characterized by the progressive degeneration of motion segments adjacent to the fusion site. The pathogenesis of ASD is believed to involve altered biomechanics and increased mechanical stress on the adjacent segments due to loss of motion at the fused level. Reported rates of ASD vary but have been documented in up to 33% of patients, depending on follow-up duration [1,2].

In an effort to preserve more natural spinal biomechanics and potentially reduce the risk of ASD, dynamic stabilization systems have been introduced. These systems employ semi-rigid materials such as polyetheretherketone (PEEK), which offer a degree of flexibility while maintaining sufficient stability. PEEK rods are hypothesized to provide a more physiological distribution of mechanical load, potentially preserving adjacent segment function [3].

More recently, hybrid systems—commonly referred to as “topping-off” techniques—have gained attention. These approaches combine rigid instrumentation with interbody fusion (e.g., transforaminal lumbar interbody fusion, TLIF) at the index level and dynamic stabilization at the adjacent cranial segment, which is at the highest risk for ASD. This dual approach seeks to ensure solid fusion where necessary while mitigating the biomechanical stress transmitted to adjacent levels [4].

The objective of this study is to compare the clinical and radiological outcomes of four different surgical stabilization techniques in patients with degenerative lumbar spine disease: (1) rigid fixation, (2) dynamic fixation, (3) rigid fixation combined with TLIF, and (4) dynamic fixation combined with TLIF (hybrid system). The comparison focuses on the incidence of ASD, pseudoarthrosis rates, patient-reported outcomes using the Visual Analog Scale (VAS), and radiological parameters including segmental and total range of motion (ROM), disc height, and foraminal height.

## 2. Materials and Methods

All procedures performed in this study were in accordance with the ethical standards of the institutional and national research committee and with the 1964 Helsinki Declaration and its later amendments or comparable ethical standards. This study was approved by the institution’s ethics committee. Informed consent was obtained from all participants included in the study.

### 2.1. Study Design and Patient Selection

This retrospective, comparative cohort study included 86 patients who underwent posterior lumbar transpedicular stabilization for degenerative lumbar spine pathology between 1 January 2017 and 1 December 2021.

Inclusion criteria were as follows: age > 35 years, diagnosis of grade 1 spondylolisthesis, absence of spinal deformity (scoliosis < 10°), radiological findings consistent with clinical symptoms, and stabilization involving no more than five vertebral levels. Exclusion criteria included prior lumbar fusion surgery, malignancy, active spinal infection, trauma, congenital spinal anomalies, or rheumatological disorders.

### 2.2. Surgical Technique and Group Classification

All procedures were performed via a standard posterior approach using polyaxial transpedicular screws and rod-based instrumentation systems. Patients were categorized into four groups according to the rod material and the use of interbody fusion:

- Dynamic Group (*n* = 21): PEEK rods without interbody fusion.
- Hybrid Group (Dynamic + TLIF, *n* = 24): PEEK rods with TLIF performed at the pathological segment.
- Rigid Group (*n* = 19): Titanium rods without interbody fusion.
- Rigid + TLIF Group (*n* = 22): Titanium rods with TLIF at the pathological segment.

**Figure 1:**
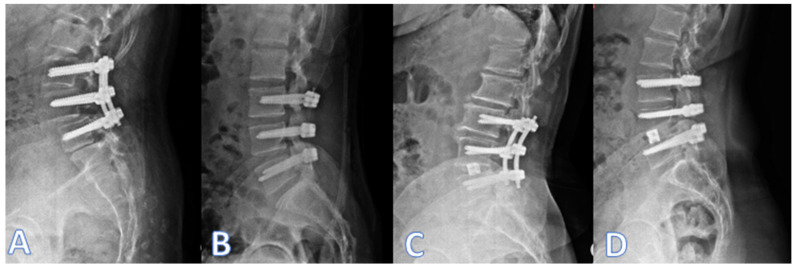
Stabilization systems exemplified on lateral direct radiographs. (**A**) Rigid Group, (**B**) Dynamic Group, (**C**) Rigid + TLIF Group, (**D**) Dynamic + TLIF (Hybrid) Group.

Patient examples are shown in Figure 1.

Because of the retrospective design, patients were not randomly allocated to treatment groups. The choice of stabilization technique was based primarily on surgeon preference and individual intraoperative considerations. Therefore, selection bias associated with treatment allocation cannot be excluded.

### 2.3. Clinical and Radiological Evaluation

Clinical and radiological assessments were performed preoperatively, during the early postoperative period, at 1 month, and at 1 year postoperatively. Radiographic measurements were obtained using standardized standing anteroposterior, lateral, and flexion–extension radiographs. In addition, all patients underwent computed tomography (CT) at the 1-year follow-up to assess fusion status and implant integrity. Anterior fusion was evaluated in the TLIF groups based on the presence of continuous trabecular bridging across the interbody space on CT. Posterior fusion was assessed by the presence of bridging bone across the posterolateral fusion bed. Pseudoarthrosis was diagnosed based on radiographic evidence of failed fusion, including the absence of bridging bone, abnormal motion on dynamic radiographs, CT findings consistent with nonunion, or implant loosening in fusion-intended constructs. In the Dynamic group, where fusion was not intended, screw loosening was recorded as mechanical failure of the construct. To minimize measurement variability, evaluations were performed according to a predefined protocol by experienced spine surgeons who were not involved in the statistical analysis.

Segmental ROM was measured on flexion–extension lateral radiographs as the angular difference between maximal flexion and extension. Total lumbar ROM was calculated similarly across the lumbar spine. Disc Height Index (DHI) and Foraminal Height Index (FHI) were measured using standardized radiographic methods based on the ratio of disc or foraminal height to vertebral body dimensions, thereby minimizing the effects of radiographic magnification.

#### 2.3.1. Clinical Evaluation

Back and leg pain intensity was assessed using the Visual Analog Scale (VAS), ranging from 0 (no pain) to 10 (worst possible pain).

#### 2.3.2. Radiological Evaluation

Standing anteroposterior and lateral lumbar radiographs, as well as dynamic flexion–extension X-rays, were obtained at each follow-up. The following parameters were evaluated:

- Segmental and total range of motion (ROM)
- Lumbar lordosis
- Disc Height Index (DHI) and Foraminal Height Index (FHI) at the adjacent segments
- Fusion status (anterior and posterior)
- Presence of pseudoarthrosis
- Development of adjacent segment disease (ASD)

Adjacent-segment pathology was evaluated using standing anteroposterior and lateral radiographs and dynamic flexion–extension radiographs obtained during follow-up. Computed tomography was performed at one year in all patients, primarily for assessment of fusion and implant integrity. MRI was not routinely obtained in asymptomatic patients but was performed in patients who developed new or worsening clinical symptoms suggestive of adjacent-level neural compression or degeneration.

Radiographic adjacent-segment degeneration was defined as newly developed or progressive imaging abnormalities at a motion segment immediately adjacent to the operated level, including disk-space narrowing or degeneration, new or progressive spondylolisthesis, facet degeneration, or spinal canal or foraminal stenosis, without necessarily being associated with symptoms.

Symptomatic adjacent segment disease was diagnosed only when a new or progressive adjacent-level radiological abnormality was accompanied by clinically concordant symptoms, including new radicular pain, neurogenic claudication, neurological deficit, or clinically significant axial pain attributable to the corresponding adjacent segment. Radiographic abnormalities without corresponding clinical symptoms were classified as adjacent-segment degeneration and were not counted as ASD events.

Imaging assessments were performed by experienced spine surgeons who were not involved in the statistical analysis. Blinding to treatment allocation was not feasible because the rod material, implant configuration, and presence of an interbody cage were visible on postoperative imaging.

Standing anteroposterior, lateral, and flexion–extension lumbar radiographs were obtained preoperatively and during postoperative follow-up. Thin-slice computed tomography was systematically obtained at the one-year follow-up in all patients and served as the primary imaging modality for assessment of fusion status and implant integrity. Flexion–extension radiographs were used as a complementary modality to evaluate residual or abnormal motion at the treated levels.

Anterior interbody fusion was assessed only in the TLIF-treated groups. Fusion was defined as the presence of continuous trabecular bone bridging across the interbody space on sagittal or coronal CT reconstructions, without a persistent radiolucent gap surrounding the interbody graft or cage. Posterolateral fusion was defined as continuous bridging bone across the posterolateral fusion bed between the instrumented vertebrae.

Pseudoarthrosis was diagnosed in fusion-intended constructs when one or more of the following findings were present: absence or interruption of bridging bone on CT, persistent radiolucency at the intended fusion site, abnormal motion at the treated level on flexion–extension radiographs, implant loosening, or implant failure considered radiologically consistent with nonunion. CT findings were considered the principal criterion, while radiographic motion and implant-related findings were used as supportive evidence.

Because fusion was not intended in the Dynamic group, absence of bridging bone was not classified as pseudoarthrosis in this group. Screw loosening, rod or screw failure, or loss of construct integrity in the Dynamic group was classified separately as mechanical failure.

### 2.4. Statistical Analysis

Statistical analyses were performed using IBM SPSS Statistics version 29 (IBM Corp., Armonk, NY, USA). Continuous variables are expressed as mean ± standard deviation (SD) and categorical variables as frequencies and percentages. Normality was assessed with the Shapiro–Wilk test. Longitudinal (preoperative, early postoperative, 1-month, and 1-year) measurements were analyzed with repeated-measures analysis of variance (ANOVA); pairwise time and group comparisons used the Bonferroni correction, and non-normally distributed variables were compared with the Kruskal–Wallis test. Between-group comparisons at individual time points used one-way ANOVA. Categorical outcomes were compared using the Fisher–Freeman–Halton exact test, chosen because of the small and unbalanced cell counts. For the principal outcomes we report effect sizes (Cramér’s V for categorical associations, η^2^ for between-group continuous comparisons, and Cohen’s d for pairwise comparisons) together with 95% confidence intervals (CIs); odds ratios with 95% CIs are reported for the key categorical contrasts. Because of the limited sample size and the small number of outcome events, multivariable logistic regression was considered statistically unreliable and was not performed. Because of the retrospective design, no a priori sample-size calculation was performed. The sample consisted of all consecutive patients who fulfilled the predefined eligibility criteria during the study period. A sensitivity analysis based on the available sample of 86 patients, four treatment groups, a two-sided α level of 0.05, and 80% statistical power indicated that the study could detect an omnibus effect size of approximately Cohen’s f ≥ 0.365 for continuous outcomes analyzed using one-way analysis of variance and Cramér’s V ≥ 0.356 for categorical comparisons. Thus, the available sample was primarily sensitive to relatively large between-group effects, and smaller clinically relevant differences may not have been detected. A two-sided *p*-value < 0.05 was considered statistically significant.

## 3. Results

### 3.1. Demographic and Baseline Characteristics

A total of 86 patients were included in the study: 21 (24.4%) in the Dynamic group, 24 (27.9%) in the Hybrid group, 19 (22.1%) in the Rigid group, and 22 (25.6%) in the Rigid + TLIF group. The overall mean age was 57 ± 10 years (range: 35–79), with no statistically significant difference in age distribution among the groups (*p* = 0.487). The cohort consisted predominantly of female patients (*n* = 61, 70.9%), and gender distribution was also comparable between groups (*p* = 0.303). The relevant information is shown in Table 1 and Table 2.

### 3.2. Clinical Outcomes

All four groups showed large, statistically significant reductions in both back and leg pain over time (*p* < 0.001 for the time effect). Mean back-pain VAS fell from 8.5 ± 1.5 preoperatively to 2.0 ± 1.6 at one year (a reduction of 6.5 points, ≈76%), and leg-pain VAS from 7.1 ± 2.1 to 1.2 ± 1.1 (5.9 points, ≈83%); postoperative scores at 1 month and 1 year were significantly lower than preoperative values in every group (*p* < 0.05). Between-group differences at one year were small and non-significant for both back-pain (η^2^ = 0.04) and leg-pain VAS (η^2^ = 0.05); the only significant between-group signal was a baseline difference in back-pain VAS (*p* = 0.020, η^2^ = 0.13), driven by lower preoperative scores in the Rigid group.

Post hoc analyses revealed significant differences in low back pain VAS scores between the Dynamic group and both the Rigid and Rigid + TLIF groups, whereas no significant differences were observed among the remaining pairwise comparisons. Adjacent segment disease was significantly more frequent in the Rigid + TLIF group than in the other treatment groups. In contrast, posterior fusion and complication rates were comparable among all groups.

### 3.3. Radiological Outcomes

#### 3.3.1. Range of Motion (ROM)

No statistically significant differences were observed between groups in the change in segmental ROM over time (*p* = 0.107). However, within-group analysis revealed a significant decrease in segmental ROM during follow-up in the Dynamic and Hybrid (Dynamic + TLIF) groups (*p* = 0.039 and *p* = 0.018, respectively), suggesting partial preservation of motion. No significant changes in overall lumbar lordosis were detected among the groups over time (*p* = 0.359).

**Figure 2:**
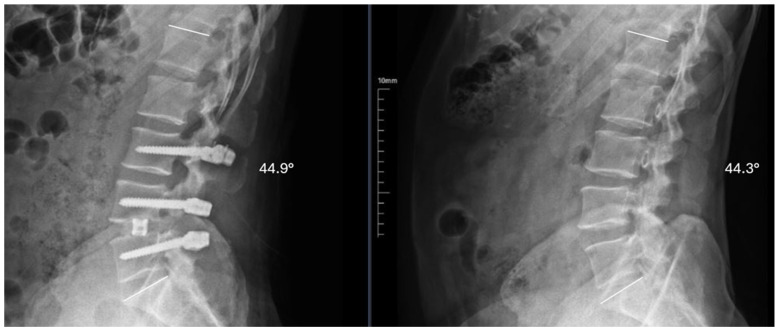
Preop–postop Total ROM (Lumbar Lordosis) measurement.

Figure 2 shows the preop–postop Total ROM (Lumbar Lordosis) measurement.

#### 3.3.2. Disc and Foraminal Height

The Disc Height Index (DHI) of the upper adjacent segment demonstrated a significant reduction over time (*p* < 0.001), with the most pronounced decrease observed in the Rigid + TLIF group at the 1-year follow-up (*p* < 0.001). No significant changes were identified in the DHI of the lower adjacent segment (*p* = 0.334). Foraminal Height Index (FHI) measurements in both upper and lower adjacent segments significantly increased in the early postoperative period compared to preoperative values across all groups (*p* < 0.001), likely attributable to surgical decompression.

**Figure 3:**
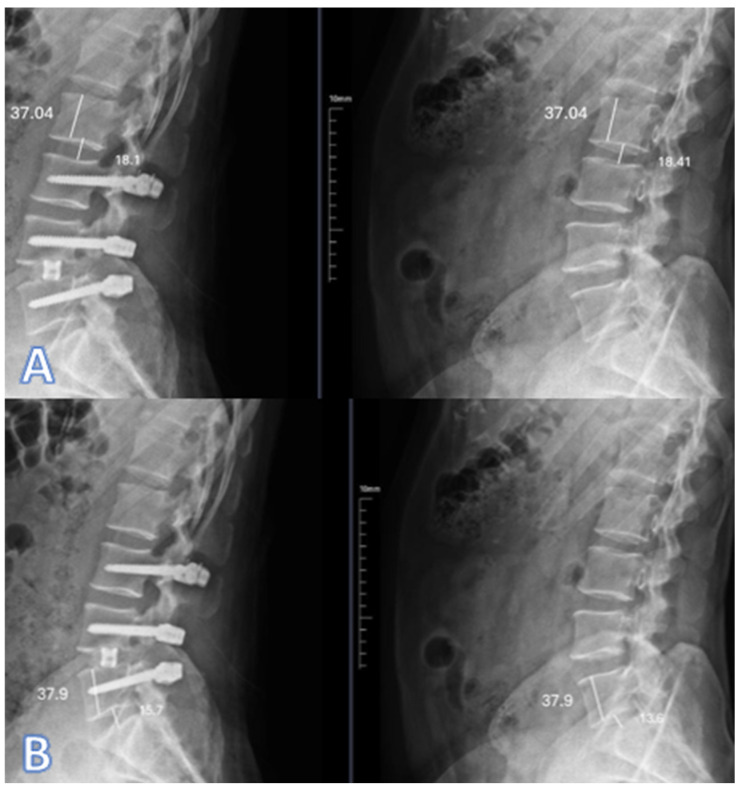
Preop–postop Upper (**A**) and Lower (**B**) disc height index measurement.

**Figure 4:**
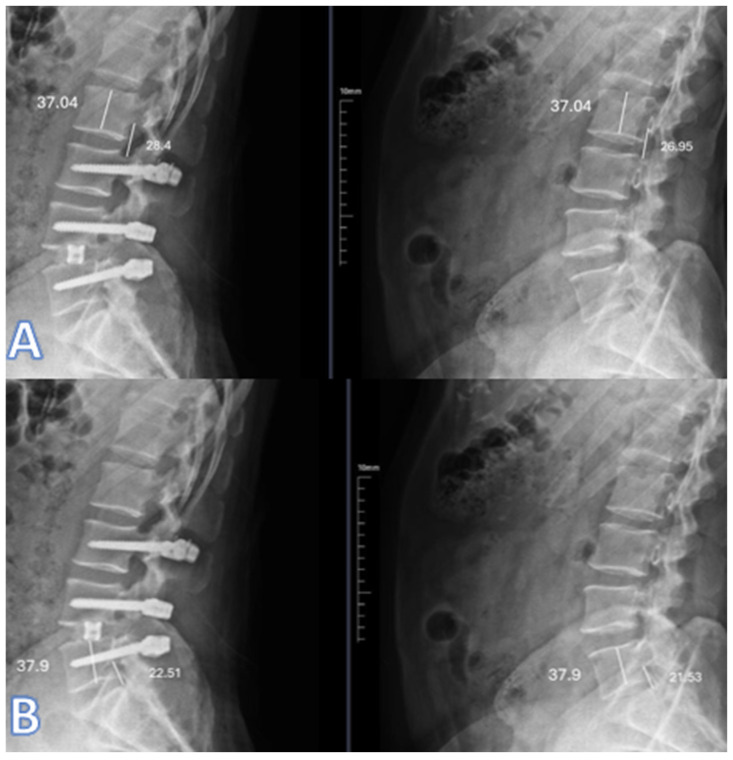
Preop–postop Upper (**A**) and Lower (**B**) foraminal height index measurement.

Figure 3 and Figure 4 shown the preop–postop Upper (A) and Lower (B) disc/foraminal height index measurement.

### 3.4. Fusion, Pseudoarthrosis, and Adjacent Segment Disease

#### 3.4.1. Adjacent Segment Disease (ASD)

**Figure 5:**
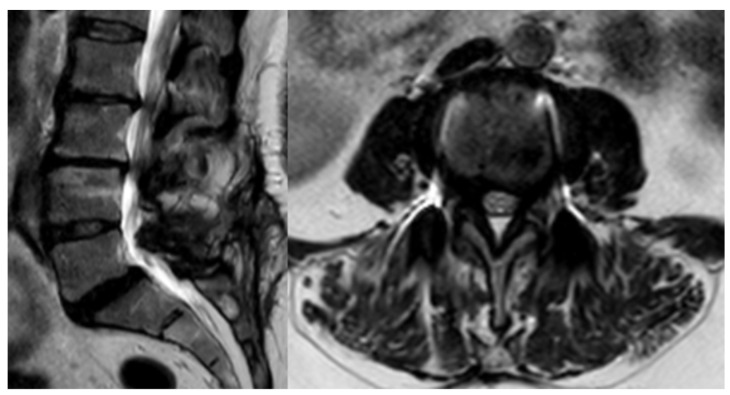
MR image of postoperative adjacent segment disease in a patient with L4-S1 rigid system.

ASD developed in 13 of 86 patients (15.1%) during the follow-up period (Figure 5) and differed significantly among groups (Fisher–Freeman–Halton exact *p* < 0.001; Cramér’s V = 0.50). The rate was highest in the Rigid + TLIF group (45.5%) and low in the Dynamic (4.8%), Hybrid (4.2%), and Rigid (5.3%) groups. Relative to all other groups combined, the Rigid + TLIF construct carried a substantially higher risk of ASD (odds ratio 16.9, 95% CI 4.1–70.9; risk difference +40.8%, 95% CI 19.3–62.2). Patients who developed ASD were significantly older than those who did not (65 ± 10 vs. 55 ± 10 years; *p* = 0.001), indicating that advanced age may be a contributing risk factor.

#### 3.4.2. Pseudoarthrosis

**Figure 6:**
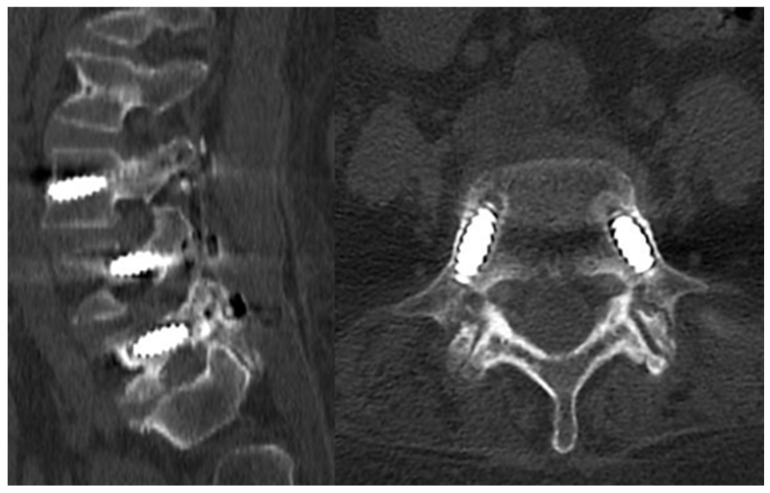
Sagittal and axial section CT image of a patient who developed pseudoarthrosis during postoperative follow-up.

Pseudoarthrosis/mechanical failure was most frequently observed in the Rigid group (26.3%) (Figure 6), significantly exceeding the Dynamic (4.8%) and Hybrid (16.7%) groups (Fisher–Freeman–Halton exact *p* < 0.001; Cramér’s V = 0.44). Relative to the Dynamic and Hybrid groups combined, the Rigid construct carried markedly higher odds of pseudoarthrosis (odds ratio 11.0, 95% CI 3.0–40.4; risk difference +46.8%, 95% CI 22.8–70.8). The absence of anterior interbody support in the Rigid construct, and the consequent loss of anterior column load sharing, is the likely explanation; the use of PEEK rods in the dynamic constructs was associated with a reduced risk of pseudoarthrosis.

#### 3.4.3. Fusion Rates

**Figure 7:**
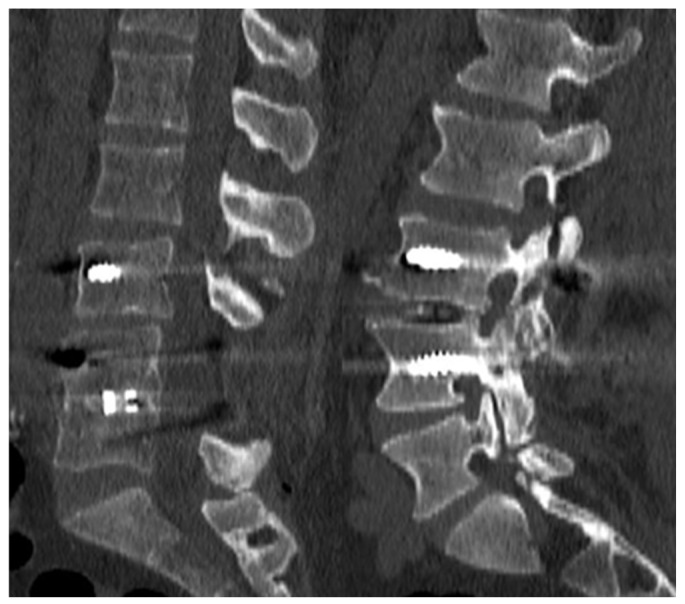
CT images of the cases with anterior and posterior fusion in the 1st year postoperatively.

Anterior fusion was achieved far more often in the TLIF-treated groups (Hybrid 91.7%, Rigid + TLIF 72.7%) than in the non-fusion groups (Dynamic 9.5%, Rigid 5.3%) (Fisher–Freeman–Halton exact *p* < 0.001; Cramér’s V = 0.76; TLIF vs. non-TLIF odds ratio 58.6, 95% CI 14.4–238) (Figure 7). Posterior fusion rates did not differ significantly among groups (28.6%, 25.0%, 10.5%, and 36.4%, respectively; *p* = 0.31; Cramér’s V = 0.21). These categorical outcomes, with exact tests and effect sizes, are summarized in Table 3.

#### 3.4.4. Complications

Postoperative complications are summarized in Table 4. Surgical site infection occurred in 5 patients (5.8%), with no significant difference among groups (Fisher–Freeman–Halton exact *p* = 1.00). No rod breakage occurred in any group. Pseudoarthrosis/mechanical failure occurred in 14 patients and was most frequent in the Rigid group, as detailed in Section 3.4.2. No cases of dural tear and cage subsidence were observed.

## 4. Discussion

Although fusion surgery remains the gold standard for addressing spinal instability in degenerative lumbar spine diseases, it may lead to long-term complications—most notably adjacent segment disease (ASD)—due to the resultant rigidity imposed on the spinal column. This study contributes to the existing literature by comparing the effects of rigid, dynamic, and hybrid stabilization strategies on the development of ASD, as well as on clinical and radiological outcomes.

When planning lumbar fusion, it is crucial to assess adjacent segment disc integrity, facet joint hypertrophy, and the degree of neural compression [5]. While rigid fusion offers immediate stability, it restricts physiological motion, which may in turn accelerate degeneration in neighboring segments. Pseudoarthrosis and ASD remain among the most concerning postoperative complications [6]. Farzam Vazifehdan et al. reported a pseudoarthrosis incidence of 7.6% in a series of 419 patients [7].

A key finding of our study was that the Rigid + TLIF group exhibited the highest incidence of ASD, despite achieving the highest rate of anterior fusion. This aligns with the hypothesis that successful fusion, while stabilizing the index segment, may increase mechanical loading on adjacent segments and trigger degenerative changes [8]. The overall ASD rate in our cohort was 15.1%, lower than the 33% reported in longer-term follow-up studies [1], likely reflecting the relatively short follow-up period in our series. Furthermore, the observation that older age significantly increased ASD risk (*p* = 0.001) highlights the need to consider patient age in surgical decision-making. Because the follow-up period was limited to one year, the reported ASD rates should be interpreted as early postoperative manifestations rather than the cumulative long-term incidence of ASD. Longer follow-up is required to determine whether these early findings persist or progress over time.

Dynamic stabilization systems, particularly those using PEEK rods, are intended to provide more physiological load sharing than rigid constructs. In the present cohort, the Dynamic and Hybrid groups had lower observed rates of early symptomatic ASD than the Rigid + TLIF group. However, this association should not be interpreted as evidence that dynamic instrumentation prevented ASD. Treatment selection was not randomized, and differences in surgical indication, baseline degeneration, sagittal alignment, bone quality, age, and the number or location of stabilized levels may have influenced both construct selection and postoperative outcomes. Therefore, the present findings support further investigation of dynamic and hybrid stabilization but do not establish a causal protective effect [3,9,10,11].

Nevertheless, given the retrospective nature of the present study and the relatively limited sample size, these findings should be interpreted with caution. It should also be recognized that unmeasured baseline differences among treatment groups may have influenced the observed outcomes. Because this was a retrospective study, several potentially important variables, including the severity of adjacent segment degeneration, sagittal alignment parameters, bone quality, and other patient-specific characteristics, were not consistently available for analysis. Consequently, residual confounding cannot be excluded, and the associations observed in this study should be interpreted within this context.

Despite concerns regarding the mechanical durability of PEEK rods, no rod breakage was observed in our cohort. Nevertheless, the higher cost and limited availability of PEEK compared to titanium remain practical drawbacks. Additionally, although PEEK rods may reduce stress shielding, they are more prone to fatigue under high-load conditions [12]. Interestingly, pseudoarthrosis was most prevalent in the Rigid group, where posterior fixation was performed without anterior interbody support. This finding underscores the biomechanical importance of anterior column support—such as that provided by TLIF—in achieving successful fusion. The lower rates of pseudoarthrosis in dynamic constructs may be attributed to controlled micromotion, which could enhance bone remodeling in accordance with Wolff’s law [13].

Radiologically, a significant postoperative increase in foraminal height index (FHI) was observed in all groups at both the 1-month and 1-year follow-ups, reflecting effective decompression of neural elements [14]. However, progressive disc height loss at the upper adjacent level was particularly noted in the Rigid + TLIF group, possibly due to altered loading mechanics in the cranial segment excluded from fusion [15]. The continuous decline in upper segment disc height across follow-ups is consistent with findings reported in prior studies.

Among the four strategies evaluated, the Hybrid system (Dynamic + TLIF) was associated with favorable radiological outcomes, including a high anterior fusion rate (91.7%) and low rates of ASD (4.2%) and pseudoarthrosis (16.7%). The “topping-off” approach, combining rigid fusion at the pathological level with dynamic stabilization of the adjacent cranial segment, may provide a biomechanically advantageous compromise between stability and motion preservation. Similar findings have been reported by Sears et al., who observed reduced reoperation rates with this technique [4]. However, because of the retrospective design and relatively small cohort, the present findings should not be interpreted as evidence of superiority over conventional stabilization methods. Rather, hybrid stabilization appears to be a promising alternative that warrants further investigation in larger prospective studies with longer follow-up periods.

Degenerative lumbar spine disease is initially managed with conservative, rehabilitation-based care, which remains the first-line approach before surgery is considered; the evidence base for these interventions in adults with spinal disorders and pain syndromes has been synthesized in systematic reviews of the Cochrane rehabilitation literature [16]. Surgical stabilization is therefore reserved for refractory cases, as in the present cohort. Moreover, because posterior instrumentation requires dissection of the paraspinal musculature—whose endurance and fatigability influence low-back pain and can be quantified with validated surface-electromyographic methods [17]—muscle- and motion-preserving strategies remain clinically relevant, and structured postoperative rehabilitation is an important adjunct to the surgical outcomes reported here.

## 5. Limitations

This study has several limitations. First, its retrospective design and absence of randomization introduce the possibility of selection bias, as treatment allocation was primarily based on surgeon preference and intraoperative considerations. Second, the sample size was relatively small, with 19–24 patients in each treatment group, and the study was conducted at a single center, which may limit the generalizability of the findings. Because no a priori sample-size calculation was possible, a sensitivity analysis was performed using the available cohort. This analysis showed that the study was primarily capable of detecting relatively large between-group effects. Consequently, small-to-moderate but potentially clinically meaningful differences may have remained undetected, and the possibility of type II errors should be considered, particularly when interpreting nonsignificant comparisons. Such findings should not be regarded as demonstrating equivalence among the treatment strategies.

Third, only 13 ASD events occurred during follow-up. This limited event count may have produced unstable and imprecise estimates, as reflected by the wide confidence intervals, and prevented reliable multivariable adjustment for potential confounders. Factors including age, baseline adjacent-segment degeneration, sagittal alignment, bone quality, and pathology distribution may therefore have influenced the observed associations. Fourth, follow-up was limited to one year, precluding evaluation of long-term implant performance and the cumulative incidence of adjacent-segment degeneration. Finally, interobserver and intraobserver reliability of the radiological measurements was not formally assessed. Accordingly, the findings should be regarded as exploratory and hypothesis-generating. Larger prospective multicenter studies with longer follow-up and appropriately powered samples are required to confirm these observations.

## 6. Conclusions

All stabilization techniques provided significant clinical improvement in patients with degenerative lumbar spine disease. Dynamic and hybrid stabilization systems were associated with lower rates of adjacent segment disease compared with rigid fixation, while TLIF-treated groups demonstrated higher fusion rates. These findings suggest that preserving spinal biomechanics through dynamic or hybrid constructs may represent a promising strategy for reducing adjacent segment degeneration. Further prospective studies with larger patient populations and longer follow-up are warranted to clarify the long-term efficacy and optimal indications of these stabilization techniques.

## Figures and Tables

**Table 1:** Demographic Data of Patients.

Variable	Dynamic (*n* = 21)	Hybrid (*n* = 24)	Rigid (*n* = 19)	Rigid + TLIF (*n* = 22)	*p*
Age (years)	56.0 ± 9.5	56.5 ± 9.8	57.0 ± 10.0	58.5 ± 10.2	0.487
Male, *n*	6	6	7	6	0.303
Smokers, *n*	4	4	5	5	~0.98
Diabetes mellitus, *n*	2	2	3	3	~0.97
Hypertension, *n*	7	6	7	7	~0.99
Osteoporosis, *n*	2	2	3	2	~0.99

**Table 2:** Baseline (pre-operative) radiological characteristics by group.

Parameter	Dynamic (*n* = 21)	Hybrid (*n* = 24)	Rigid (*n* = 19)	Rigid + TLIF (*n* = 22)	*p*
Segmental ROM (°)	28.0 ± 3.4	29.5 ± 3.1	28.8 ± 3.5	29.2 ± 3.3	0.47
Lumbar lordosis—total ROM (°)	48.9 ± 14.0	54.5 ± 39.6	48.0 ± 16.6	53.9 ± 16.6	0.76
Upper disc height index	0.37 ± 0.09	0.35 ± 0.08	0.36 ± 0.07	0.35 ± 0.09	0.83
Lower disc height index	0.30 ± 0.13	0.28 ± 0.07	0.28 ± 0.12	0.33 ± 0.09	0.34
Upper foraminal height index	0.69 ± 0.10	0.64 ± 0.09	0.61 ± 0.15	0.67 ± 0.10	0.12
Lower foraminal height index	0.53 ± 0.09	0.51 ± 0.08	0.48 ± 0.14	0.54 ± 0.12	0.32

**Table 3:** Categorical outcomes at one year, compared with the Fisher–Freeman–Halton exact test, with effect sizes.

Outcome (1 Year)	Dynamic (*n* = 21)	Hybrid (*n* = 24)	Rigid (*n* = 19)	Rigid + TLIF (*n* = 22)	Exact *p*	Cramér’s V
Adjacent segment disease	1 (4.8%)	1 (4.2%)	1 (5.3%)	10 (45.5%)	<0.001	0.50
Pseudoarthrosis/mechanical failure	1 (4.8%)	4 (16.7%)	5 (26.3%)	4 (18.1%)	<0.001	0.44
Anterior fusion	2 (9.5%)	22 (91.7%)	1 (5.3%)	16 (72.7%)	<0.001	0.76
Posterior fusion	6 (28.6%)	6 (25.0%)	2 (10.5%)	8 (36.4%)	0.31	0.21

Values are *n* (%).

**Table 4:** Postoperative complications, compared with the Fisher–Freeman–Halton exact test.

Complication	Dynamic (*n* = 21)	Hybrid (*n* = 24)	Rigid (*n* = 19)	Rigid + TLIF (*n* = 22)	Exact *p*	Cramér’s V
Surgical site infection	1 (4.8%)	2 (8.3%)	1 (5.3%)	1 (4.5%)	1.00	0.07
Rod breakage	0	0	0	0	—	—
CSF Fistula	0	0	0	0	—	—
Reoperation	0	0	1	1	—	—
Pseudoarthrosis/mechanical failure	1 (4.8%)	4 (16.7%)	5 (26.3%)	4 (18.1%)	<0.001	0.44

Values are *n* (%). Adjacent segment disease is reported in Table 2.

## Data Availability

The datasets generated during and/or analyzed during the current study are available from the corresponding author on reasonable request.
